# Beauty Consumption Matchmaking Mechanism for Confirming the Requirement Specification of App Development in the Post-COVID-19 Era

**DOI:** 10.3389/fpsyg.2022.925905

**Published:** 2022-06-03

**Authors:** Yang-Wen Chang, Yen Hsu

**Affiliations:** The Graduate Institute of Design Science, Tatung University, Taipei, Taiwan

**Keywords:** COM-B model, app platform requirement, beauty app, COVID-19, matching mechanism

## Abstract

COVID-19 began to spread worldwide in early 2020. Various governments have taken measures such as isolation, travel bans, and evacuation, mandating people to wear masks and go out less, in an attempt to prevent the spread of the virus. Governments also restrict human contact service industries, including beauty and hair salons. When the pandemic was very serious, consumers had great doubts about going for hairdressing so the beauty industry was greatly affected. This study designed and developed an app platform that considers the COVID-19 and is used for the psychological role of consumer safety. The methodology of this research is a qualitative study. Based on the Capability, Opportunity, Motivation, Behavior (COM-B) model, and presenting the context of capabilities, opportunities, and motives of the theoretical framework, this study investigates the factors that identify the demand for the development of the platform for the beauty industry app matching mechanism. Four groups of people including consumers, beauty technicians, store managers, and application development engineers all conduct interviews based on their ability, opportunity, and motivation after experiencing the using application of OpenBeautiful. The results found that all groups of people already had the knowledge and ability to avoid infection in a pandemic, so it was possible to establish the need and feasibility of using the app platform. The use of the beauty industry app platform can bring new consumption patterns and career opportunities, and can trigger more business behavior activities. Therefore, this study starts from the COM-B model, and then explores the user needs of the platform according to the COM-B model framework, and proposes the mechanism and platform of app matching. Finally, based on the conclusion of the study, we propose practices and suggestions for the future operation of the app matching platform.

## Introduction

COVID-19 was discovered in January 2020 and has spread to nearly 200 countries worldwide (Fahmi, 2019; Zhu et al., 2020). Every country around the world is suffering a great impact (Baldwin and Tomiura, 2020). The government encourages people to stay at home, and restricts most of the service industries, including beauty, hairdressing, salons, beauty clinics, and other human contact services, thus affecting the survival of all practitioners. In addition, the COVID-19 epidemic has led to negative consumer stagnation among beauty service users, and the pandemic has led to an increase in life stress and a loss of self-control mechanisms among the general public (Tan et al., 2020). It also creates many unpredictable and uncontrollable sources of stress in daily life (Pfefferbaum and North, 2020).

Many people rely on beauty-related treatments such as nail, hair, and face care in their daily lives to increase self-confidence and reduce psychological stress in their lives (Back and Chang, 2012). There are also non-surgical and cosmetic surgical methods to increase self-confidence (Castle et al., 2002; Margraf et al., 2013; Oates and Sharp, 2017). So for some people, seeking beauty-related services can help relieve their anxiety about appearance, which is very important for individuals (Veale, 2004; Wilhelm et al., 2013). Therefore, cosmetic industry services are one of the methods that can improve and relieve stress due to COVID-19 panic (Pikoos et al., 2020).

During the COVID-19 pandemic, people’s habits changed to a home-based lifestyle, with an increase in exercise and eating activities at home (Phillipou et al., 2020). Before the COVID-19 epidemic, many people often went to stores to make themselves more beautiful before going out to work and socialize, but now they have to do beauty treatments themselves mostly at home to avoid contact with others. Such changes have seriously affected the consumer market and employment opportunities in the beauty industry (Neziroglu et al., 2008; Radix et al., 2019). In response to the impact of covid, the beauty service industry is challenged to survive and must adopt a different approach to deal with it (Lunn et al., 2020). Now everyone has a cell phone. People like to use the app on the phone because it is convenient and easy to use. Therefore, under the impact of the covid epidemic, the solution is to design a mobile app service. To encourage consumers to maintain their previous habits and still be safe first of all, the app designed a reservation service interface function. This function can be sure to help consumers find safe beauty technicians and beauty stores easily. Through this reservation function, we can check whether the consumer is safe, the beauty technician is safe, and the store is safe before the beauty service is carried out, which can be achieved in the least time and efficiently. After the service is completed, the app can also provide a convenient and secure cash flow system to complete online payment collection.

OpenBeautiful app is a small business innovation and research (SBIR) grant program of the Small and Medium Enterprise Division, Ministry of Economic Affairs, Taiwan, and is the three-party appointment service app platform for the beauty industry. The design of the mobile network app platform interface, to achieve the consumption function of the reservation service. In this study, the OpenBeautiful app platform was used to conduct stages of formative research, testing, and iteration (White et al., 2016). To let users feel in the test, we hope to seek design and usage suggestions and feedback through the operation in the test. Beauty services boost consumer confidence related to consumer behavior (Michie et al., 2011). Under the influence of COVID-19, integrated business practices that require human contact need to be transformed in the pandemic’s daily life (Banca et al., 2020). People’s behavioral changes due to the prevention of infection are also the reason why society is facing many urgent problems. Furthermore, the COM-B theoretical model provides a research framework for analyzing the factors influencing people’s behavior change. Therefore, this study uses the COM-B model as a research framework to form a research model for understanding mutual intervention relationships based on capability, opportunity, and motivation. User testing of the OpenBeautiful app platform first, followed by theoretical support for the topics discussed in the focus interviews (McClelland et al., 2017). The results of the study are expected to identify the requirements of users for app platform development. Understand what users expect from the platform features, what they consider when using the platform, and the relationship-building or promotion benefits the platform can bring in the future to better manage the operation and marketing of the app.

## Literature Review

### Beauty Industry: Definition, Characteristics

The existence of the beauty industry is the actual meaning of people’s pursuit of beauty. Influenced by the culture of celebrities and global media, the culture of different countries, nationalities, or individuals differs, and it is an indicator of fashion and taste in consumer behavior, and at the same time, it also covers the subjectivity and specificity of personal taste (El Jurdi and Smith, 2018). There is a strong emotional connection between beauty industry consumers, beauty products, and beauty industry workers. A beauty technician is a person who handles all kinds of beauty treatments for customers, including skin, face, eyelashes, eyebrows and makeup, manicure or hair styling, and other practice items. Beauty store owners are physical store operators. They can perform business related to the beauty industry. They hire some beauty technicians to serve consumers in physical stores. The above service staff and beauty stores by the Taiwan government regulations, the beauty industry must pass the examination before they can launch their business.

Beauty services include skill-oriented services, including beauty salons, studios, SPAs, fitness centers, and other venues (Chan et al., 2017). Medical-oriented aesthetics include skin treatment at medical aesthetic centers and invasive treatments such as cosmetic surgery, weight loss, and scar removal. Sales-oriented beauty merchandising services can be divided into merchandise advertising, physical merchandise and beauty industry equipment, and other channels of consumer orientation.

As far as the service characteristics of the beauty industry are concerned, most of the services are provided by technicians who perform their professional skills at close range and gain a sense of trust with consumers in the mode of interaction with customers (Chan et al., 2017). Bateson (1985) proposed that consumer service is an experience feeling and context of the service, the consumer is served by the experience value and message to decide whether to buy the service again. In addition, most of the technicians in the beauty industry and customers are in contact with each other. Therefore, the technicians provide good products and services to each other and the customers in a good consumer atmosphere, will book the next beauty service (Leung et al., 2019). The labor-intensive service industry does not have production, distribution, and logistics like physical shopping malls, because it has talent deployment or manpower matching to meet customer demand. So, the shared media economy of goods has shaken the traditional service industry (Ma et al., 2021). Since the service connotation of each beauty technician or each physical beauty store cannot be formatted, the feelings of each customer are not the same. Although the practitioners then provide services to create differentiated and distinctive services, the attitude of improving service quality has always been the common goal of operators (Pine and Gilmore, 1999).

### New Consumption Patterns, New Normal (COVID-19)

In the past, typical consumer perceptions depended on the consumption of agricultural or industrial products, services, and housing (Grundey, 2009). Consumer behavior is the decision-making process of searching for information about a product or service, buying it, using it, evaluating it, and continuing to buy it (Valášková and Klieštik, 2015). Macroscopic consumer behavior is caused by social issues, but in order to reach the microscopic factors of consumer behavior, individual factors are studied. Flatters and Willmott (2009) said Consumers, try to maximize their utility, satisfaction, or happiness by purchasing consumer goods.

During the COVID-19 pandemic, demand for home care, cosmetic and personal care products declined. Issued in May 2020 “How COVID-19 is changing the world of beauty” (Gerstell et al., 2020), a survey was conducted on consumer sentiment during the crisis. The results were that 70% of the consumers in the sample strongly agreed to be careful with their spending and reduce their purchases. The beauty industry is also increasing online business sales services as consumers change their consumption behaviors. This pandemic has had a serious impact on the country’s economy and has also produced changes in market dynamics. In the report on market trends and D2C opportunities in COVID-19, Shinzo Abe observed this trend. For example, during a pandemic, people spend less of their income on items that are considered already haves or non-essentials such as clothes, shoes, cosmetics, jewelry, games, and electronics. Higgins et al. (2020) mentioned keeping a social distance is now widely considered to be the best way to control the spread of viruses. The use of AI, cloud computing, and always-on independent device software functions to reduce human contact, can block viruses and efficiently allow consumers to feel safe and hygienic. COVID-19 has had a serious impact on the global economy since March 2020 (Baldwin and Di Mauro, 2020; Baldwin and Tomiura, 2020), With the uncertainty of the COVID-19 outbreak, social networks are an important tool for the general public to get information (Westerman et al., 2014). After the COVID-19 epidemic, consumers are affected by the financial ability of personal consumption, but it may provide a new change in consumption habits involving personal health care and self-care behaviors, and such a change will generate new values and bring a new type of consumption (Anderson and Wadkins, 1991). Online matchmaking platforms can provide consumers with contactless hairdressing appointments, reducing the risk of infection and increasing consumer willingness.

As COVID-19 moved from local to global outbreaks, there was a growing concern about the practicality of consumption as people began to cut back on unnecessary spending to save money. In addition to changing consumer spending patterns during the pandemic, the use of e-commerce application platforms was extensive. Consumers are not leaving their homes to shop for essential products on official websites such as social media and related products. This trend is reflected in the number of apps that consumers download for media, entertainment, medical information, and lifestyle information (Accenture, 2020). These platforms are expected to play a significant role in changing consumer patterns after COVID-19 by spreading word-of-mouth, product awareness, transactions, and retaining consumer interaction (Deloitte, 2020).

### COM-B: Theoretical Basis

**FIGURE 1:**
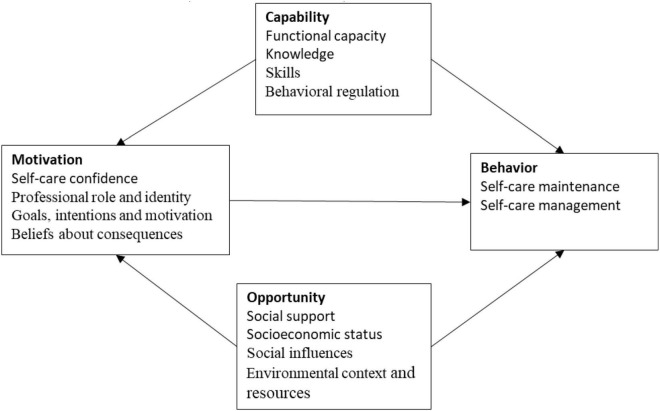
COM-B research conceptual model.

In 2005, Michie established a theoretical framework for 12 domains by searching for consensus among 33 organizational theories and psychologies (Cane et al., 2012). In 2011, Michie proposed the COM-B model of behavior change theory, the main components of which include capability, defined as the mental or physical ability to engage in an activity that includes the knowledge and skills that an individual possesses. Opportunity is defined as all factors that have an impact on society or individuals other than those that allow the behavior to contribute to its occurrence. Subjective motivation is defined as a reflective self-mechanism that includes self-efficacy and degree of emotional arousal (Figure 1). In the framework of the behavioral system, the COM-B model presents human behavior as interacting with an individual’s objective capabilities, opportunities, and subjective motivations, with the three cores influencing human behavior through reflexive action instead (Michie et al., 2011). Behavior change theory is a theoretical approach to understanding patterns of behavior. The design process can be efficiently increased and the objectives can be effectively changed during the process (Michie et al., 2005).

The COM-B model is widely used in health interpretation and prediction analysis (Francis et al., 2012). An example from a study of a family-centered care disability and hearing practice by a private hearing healthcare company in Australia. The COB-B model was used to explore employees’ perspectives, and the study found that different employee bases have different views. The study, which interviewed 13 participants including managers, clinicians, and staff, found that some behaviors must be tailored to address behavior change (Ekberg et al., 2020). A qualitative structured interview using the COM-B model was designed to identify the behavioral program determinants of audiologists in a study to improve hearing aid restoration in adults (Barker et al., 2016). A qualitative study used semi-structured in-depth interviews for the development of an e-health care system for families of children with hearing loss. The theme of the interview was to explore opportunities, capacity-related barriers, and facilitator motivations using the COM-B model of behavior change (Nickbakht et al., 2020). This study used the COM-B model and theoretical framework to examine the mediating role of self-care confidence in exploring factors related to self-care behaviors. It has shown that confidence in self-care is mediated by the relationship between knowledge, health literacy, social support, and self-care behaviors (Zou et al., 2017). To improve some of these situations, research is optimized for practice, developing life-changing science and technology to design interventions that will enhance improvements and make program policies useful to people. The 12 frameworks derived from the three perspectives of the integrated COM-B model were used to conduct the focus interview spine to understand the different dimensions of relevant behavior change. This study is based on the content of the framework interviews to explain behavioral patterns and predictive analysis and applies the method to identify the functional requirements of the beauty app development platform. In this study, the COM-B model was used to identify the knowledge, skills, behavioral regulation, and functional abilities under the capability construct. Under the opportunity framework, there are social support, socioeconomic status, social influence, environmental background, and resources, respectively. Under Motivation, there are Self-Care Confidence, Professional Role and Identity, and Goal Intention and Motivation.

### App Functions

**FIGURE 2:**
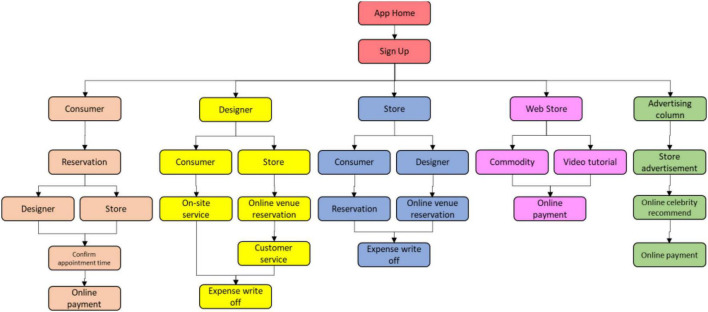
OpenBeautiful app function structure.

The main function of the membership management is the member login and history management, and there are three types of roles, which are shop managers, beauty technicians, and general consumers. There is also basic customer information, basic product information, arrival service records, and search functions. The reservation system allows consumers to choose the appointment time and venue. Consultation systems can provide a platform for members to chat with each other. Consumers can rate service providers with stars in the rating system and have the ability to track favorites to increase opportunities for more services opportunity. System administrators can make good use of the reservation management and membership management system to improve the service capability of the system, and the advertising management can increase the source of income, exposure, and public welfare message for the system members. SMS text messaging system linked to the telephone company, which allows consumers to receive a successful booking message quickly. The system function includes electronic invoice management, providing consumers with a record of the amount spent. In addition, shop managers have a coupon management system to increase the incentive to spend, a management system for tandem sales verification, third-party payments and orders, and a refund system mechanism that allows consumer-members to cancel appointments for refunds. OpenBeautiful app function structure is shown in Figure 2 below. Please see Appendix I for detailed functions.

## Research Design

**FIGURE 3:**
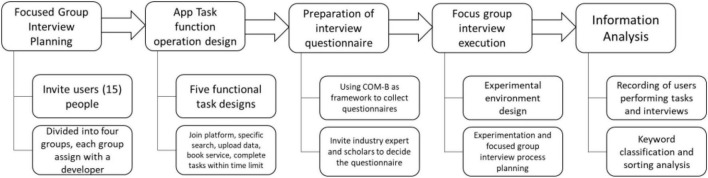
Research procedure.

This study was conducted to confirm the OpenBeautiful app platform design and development. The general consumers, beauty industry designers, and beauty industry stores were invited. They did task operation, operation items, and process of beauty platform app respectively as Figure 3 shown.

### Research Procedure

In order to explore the differences in users’ knowledge behaviors, group interviews were conducted to better present the attitudes and experiences, as well as the elements of specific behaviors to complete this study (Kitzinger, 1995). Participants were asked to complete a brief demographic survey to understand their background information. After using the test version of the app, the main content is based on the customer contact process, customer contact and service process, and comments on the relevance of the use of the app to the customer. The research team recruited 15 women to conduct three focus groups with general consumers, beauty industry designers, and beauty industry stores, with a sample size of five in each focus group. Among them, beauty designer members and beauty store managers must have been practicing beauty for at least six months to ensure the acceptability of the survey results and participation strategy. Each focus group has a technical person with app programming skills to assist with interviews or operations. After the interview, we must also leave the interview guide notes for the research team to check the records and provide confirmation of customer requirements for app development. We also provide feedback and suggestions after use to maintain customer-oriented service design and development.

### Open Beautiful App Development of Prototype Phase Testing

**FIGURE 4:**
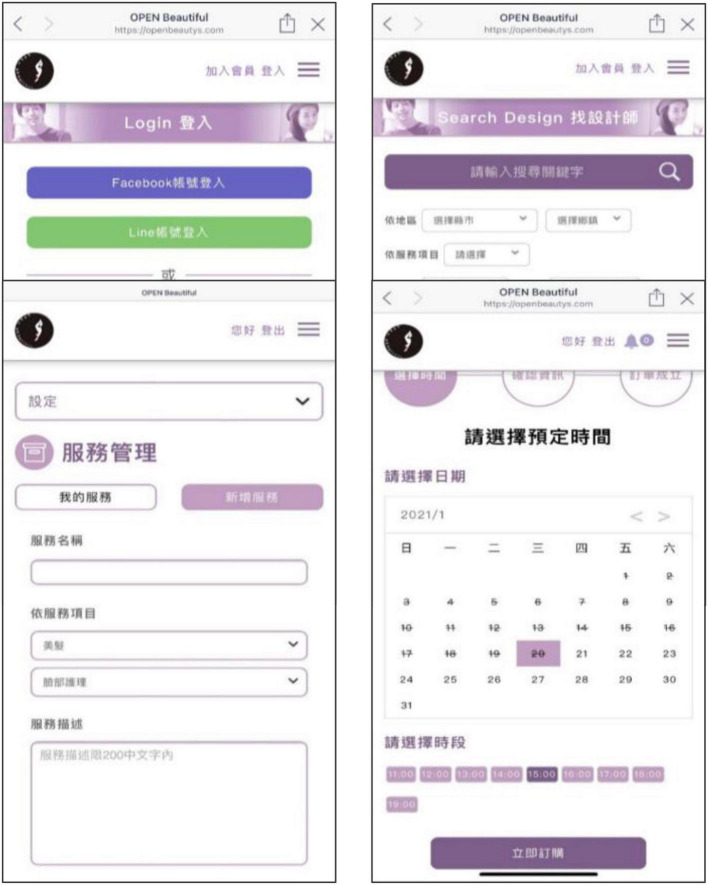
OpenBeautiful app user interfaces.

The general consumer, beauty designer, and beauty store manager each performed tasks on the beauty platform app provided by the testers, including joining the platform, specific search, uploading information, booking services, and completing the tasks within a fixed time frame. Feedback operations allow test subjects to check for software defects, visual and user experience, or other issues in successive iterations of the application, in the hope of seeking feedback on design and functionality through such procedures and habits. OpenBeautiful app user interfaces are shown in Figure 4 below.

### Interview Questions

Many scholars using COM-B have been characterized by cross-validation using the Theoretical Framework for Behavioral Change (Cane et al., 2012). Including the derived framework structure behavior, including theories of motivation, action, social cognitive theory, organizational theory, and social influence (Michie et al., 2005) proposed. These structural theories can be applied to the COM-B system framework in a convergent manner and allow researchers to apply COM-B systematically to questionnaire design for qualitative, quantitative, and quantitative analysis. For example, Varisco et al. (2020) used the original COM-B constructs as the interview infrastructure as a reference for adapting their interviews from questionnaires from different locations.

In this study, industry experts and scholars were invited to collect relevant topics from existing literature and conduct expert interviews in the following three steps:

(1) Topic preparation: Through literature collection and classification, the following questions were obtained.
(2) Invite experts: 5 experts were invited for this study, one lecturer with more than 10 years of experience in the beauty industry association and one person in charge of an app development company with 20 years of experience, 1 university professor with 20 years of experience in questionnaire design, 1 university professor with 15 years of experience in statistics, 1 university professor with 20 years of experience in information management.
(3) Expert Interview Process:
  A. Introduction to our study.
  B. Ask the expert to answer.
  C. Statistical Analysis.
  D. Discuss the decision.

The results of the interviews are structured in COM-B, with the capabilities, opportunities, and motivations divided into four constructs. For each construct, two questions are provided as listed in Table 1 below.

**TABLE 1:** Focused interview group questions based on the COM-B model.

Construct	Sub-construct	Interview questions	References
Capability	knowledge	1. Do you have a basic understanding of basic COVID-19 infection prevention? (e.g., mask, safety distance). 2. Do you have any suggestions for beauty app business projects?	Plohl and Musil, 2020
	Skills	1. How do you think the training should respond to the market demand for functional training or functional self-improvement in the beauty industry? 2. Can you access and analyze the needs of consumers?	Chen, 2015
	Behavioral regulation	1. If beauty technicians are independent of the store, how do you think consumers can distinguish their role? 2. What do you think about paying a service fee if you use the app booking service platform?	Hsu and Lin, 2014
	Functional capacity	1. Do you have any comments on the use of the current app function for food, clothing, housing, and transportation in the market? 2. What are your needs for the search function of a mobile app?	Al Qeisi and Al-Abdallah, 2014
Opportunity	Social Support	1. What do you think is the best way to design a three-party appointment booking service platform to facilitate job opportunities? 2. What kind of interface features do you think should be available on the COVID-19 for the three-party appointment booking service platform?	Mehta et al. 2020
	Socioeconomic Status	1. Do you feel that there are some new patterns of consumption habits during the COVID-19 pandemic? 2. What are your concerns about the COVID-19 epidemic that affects your consumer preferences?	Paredes et al. 2020
	Social Influences	1. What do you think is your preference for this kind of appointment service in the beauty industry under the influence of the COVID-19 epidemic? 2. During the epidemic, what do you think are the ways for people to protect themselves from hygiene practices?	
	Environmental context and resources	1. Do you have your own control method to prevent mass infection? 2. Because of the epidemic, we have developed an app that allows us to make appointments for beauty industry services at rented temporary sites. Do you agree it is a good idea?	CDC, 2020
Motivation	Self-care confidence	1. Would you consider the risk of exposure when going out during the COVID-19 outbreak? 2. How do you assess and prevent risk during the COVID-19 outbreak?	Plohl and Musil, 2020
	Professional role and identity	1. How do you interact with your old customers or former service staff to maintain relationships because of the outbreak? 2. How do you think you can build brand image and awareness in the app platform?	Pikoos et al., 2020
	Beliefs about consequences	1. How much did you change your daily routine during the outbreak? 2. How do you see the need for online communication in the beauty industry during the COVID-19 outbreak?	
	Goals intentions and motivation	1. Where do you think there is a market incentive before building an app beauty industry three-party booking service? 2. In the environment of lower consumption, do you think the app media on the consumer side, what do you think needs attention?	Mehta et al. 2020

### COM-B Focus Group Interview Guide

Interview guide based on COM-B behavior change theory to facilitate focus group interview discussions (Table 1). The way it was conducted began with an introduction to the purpose and process of this focus group interview. Each member is then given a focused interview guide with basic information and a written consent form. Members must sign the consent form to allow future scholarly publication of this interview study. Next, each member is asked to fill in basic information such as age, gender, occupation, title, years of experience, education, income, contact information, etc. Focus group interviews are then conducted. The entire process will be recorded. Members may also fill in comments on the interview guide, and all completed information will be collected after the meeting for analysis of the interview. In addition, pre and post-interview reports were emailed to each participant for further feedback.

## Research Results

### Experimental Process

When the group discussion began, each group member asked each other to wear masks and keep a social distance of about 1.5 meters apart. Because of this, another meeting room was opened for further discussions and interviews, allowing participants to immediately feel the tension of the COVID-19 outbreak. First of all, the moderator explained the main purpose, content, and execution process of this experiment. Four tasks were divided into groups, followed by group discussions, each group consisting of an app development engineer, consumer members, beauty technicians, and store representatives. Among them, the beauty industry is represented by professional technicians. The process is divided into two stages, one is to complete the app tasks, including joining the platform, specific search, uploading data, booking services, and setting a fixed time to complete the operation tasks above. Second, the group discussion was followed by a pre-determined question in which members exchanged information with each other and the process was recorded and converted into a verbatim transcript, which was then interpreted article by article by the researcher to find the keywords and count the number of occurrences in the period. The results of the interviews are grouped as in the Focused Interview Group Assignment Table 2.

**TABLE 2:** Focus group assignment table.

Focus group	Group 1	Group 2	Group 3	Group 4	Group 5
Software Engineer	A1	A2	A3	A4	A5
Consumer	B1	B2	B3	B4	B5
Store manager	C1	C2	C3	C4	C5
Beauty technician	D1	D2	D3	D4	D5

### Interview Content Analysis

The interview results of each question were organized and presented in the following tables. For each question, all the respondents’ answers were organized together and the keywords of each question were identified and the number of occurrences was summed to represent the importance of the keywords. Secondly, this study also describes the key points of each question and lists the content of several interviewees’ conversations.

The first question of this study can fully demonstrate that the participants have knowledge of COVID-19 infection prevention. We all have the ability to wear masks and avoid group gatherings in confined spaces and other protection, thus causing people to be reluctant to go out and spend money, which can embody the opportunity to use app in the epidemic and enhance the usage rate, thus the opportunity to create a business model for app in the beauty industry.

*Because COVID-19 is highly contagious, it is necessary to reduce the number of trips and gatherings of people, wear a mask when going out, and disinfect the whole body immediately at home. [Consumer Member B1].*

*Self-management, frequent hand washing, alcohol disinfection, wearing a mask to prevent the risk of droplet infection, and keeping a social distance. [Store Member C1].*

Because each focus interviewer’s role is different, they are very creative in the business projects they propose during the interview, and they also pay attention to the applicability of their workplace skills. However, since the number of keywords is very evenly distributed, it can be understood that the discussions and interviews show a wide range of opinions, divergent views, and the need to use the interface with multiple functionalities.

*The matching mechanism, ratings, discount codes, and member bonus points can increase the interactive effect of app members. [Engineer A2].*

*There are 3 points from Consumer Member B3. 1. Members get rebates for spending 2. Consumers get bonuses for referring customers to expand their customer base 3. Technician “studio location map” clear statistics. [Consumer Member B3].*

In order to understand whether the beauty industry workers can keep up with the ability of app reservation platform users in the application of their functions. Tables 3–6 collate the results of the interviews and the cumulative number of keyword occurrences. In the post-task interview of this study, it was found that most of the respondents agreed that in order to meet the market demand, they need to improve their skills and management abilities through vocational training and further education. Therefore, it is feasible to use the app design service in the market. The beauty industry workers have the ability to use it for their functions.

**TABLE 3:** Interview results I.

Question	1. Do you have a basic understanding of basic COVID-19 infection prevention? (e.g., mask, safety distance.)	Number of occurrences
Keyword	mask	18
	wash hand	9
	Avoid clustering	9
	Keep social distance	7
	Go out less	6
	Alcohol sterilization	4

**TABLE 4:** Interview results II.

Question	2. Do you have any suggestions for beauty app business projects?	Number of occurrences
Keyword	Bonus (Points) Discounts	6
	Market Demand	6
	Map Search (area)	5
	Matching mechanism	5
	Rating (Points)	4

**TABLE 5:** Interview results III.

Question	3. How do you think the training should respond to the market demand for functional training or functional self-improvement in the beauty industry?	Number of occurrences
Keyword	Vocational Training	12
	Popular Information	5
	Market Demand	5
	Beauty Industry Expertise	4
	Accumulated Works	3

**TABLE 6:** Interview results IV.

Question	4. Can you access and analyze the needs of consumers?	Number of occurrences
Keyword	Convenient and fast	6
	Service content, consumer needs	6
	Good service	5
	Inexpensive price	4

*According to the market and customer demand, we increase our professional skills and improve our skills at any time to seek new and better. [Consumer Member B3].*

*You can first go to the store to practice, increase the work experience, the future development will be more helpful. Furthermore, it is very important to use the course of study to show your self-worth. [Technician Member D5].*

The general perception of the study’s operators is that consumers want fast, inexpensive, and diverse services with a choice of services. Therefore, during the pandemic period, in addition to security concerns, maintaining consumer service content and satisfying consumers’ ability to evaluate usage are important to service concepts when designing app-related interfaces.

*Consumers want to use the app to match them with the convenience of being close to their homes.*

*[Store Member C1].*

*This is a consumer-oriented business. As long as it is convenient and of good quality, there will be business opportunities. [Technician Member D5].*

## Conclusion and Discussion

This study is to investigate the requirements validation of app media mechanism platform development under COVID-19. The COM-B model was used as the basis of the research framework to investigate the validation of the Open Beautiful app platform development. The research framework consisting of ability, opportunity, and motivation interfere with each other to influence behavior, and the app interface is tested to make users feel so that the operating procedures and habitual use are suggested and fed back for the purpose. This study is based on the contents of the focus group interviews, and the final analysis will be used as the basis for identifying the requirements for app platform development.

### Contribution

COVID-19 the epidemic has spread around the world and people have changed their living habits at home. Due to the various restrictions brought about by the epidemic, life has been deeply affected and people in the beauty industry have seen their employment opportunities and income decrease. The services of the beauty industry can bring people familiar and secure behavior patterns and soothe negative emotions during the stress of the epidemic. Therefore, we designed a beauty app platform that can take into account the need for safety in life due to the epidemic, and offer it to consumers who pursue beauty and improve their self-confidence, as well as increase job opportunities. In spite of the epidemic, the online communication between consumers and beauty technicians to confirm the safety and service content of the infected consumers and beauty technicians before booking the services through the app has achieved effective time control among consumers, beauty technicians, and beauty stores on the app booking platform.

#### Capability Confirmation in the COM-B Model Framework

In terms of confirming the capacity of the beauty industry apps, it is clear from the results of this study that people have the knowledge and capacity to protect themselves from infection after the COVID-19 outbreak for some time, and that a “no-touch” business model is in demand and acceptable in an epidemic. In addition, from the perspective of professional capability, there is no barrier to the application of the ability to use the app. As for the interface function of the app, users are concerned about the speed, convenience, and variety of service options. Secondly, in the demand of interface search function, the function of an area map and clear classification meet the user’s locality and convenience. Finally, the results of price comparison and real-time search are in line with Accenture’s, 2020 research. In addition to satisfying the security concerns of app users, it also facilitates the ability of platform users to evaluate the demand for services.

#### Opportunity Confirmation in COM-B Model Framework

During the COVID-19 pandemic, the widespread use of the application’s features also led to other online shopping opportunities for materials demanded by the beauty industry or for women’s beauty products. Thus creating new consumption patterns due to the convenience of the Internet, a result in line with Deloitte’s, 2020 research. During this study, it can be understood that people use a system of appointment selection as a beauty convenience service. At the psychological level, there is also a need for a sense of safety and trust in the environment, and the need for beauty does not change because of the epidemic. The results of the research and analysis show that the use of the beauty industry app three-party booking platform helps the beauty industry workers’ career opportunities. At the same time, it is a very good marketing platform that can be used to increase exposure and visibility by using a common platform. The rental of space in the service can help those in the beauty industry who cannot afford to open a store to get a job, and also facilitate the use of resources of existing stores. Therefore, from the above research, we can understand and confirm that the three-party booking platform in the beauty industry can create opportunities for both service providers and service recipients on the app membership platform. Such preventive measures can satisfy the perceived needs of both parties for environmental security through the design of the app. Especially, after adding online reservations and online payment, the convenience should also take into account the safety of payment and the design of refund and cancellation service mechanism, which will become the new norm in life in the future in response to such consumption habits.

#### Motivation Confirmation in COM-B Model Framework

The study revealed that during the COVID-19 pandemic, people were using a wide range of application software functions, and the more people did not gather or leave their homes, the more they needed remote communication platforms. At the same time, we will pay special attention to the news and information on epidemic prevention and restrain ourselves for the sake of epidemic prevention. However, in anticipation of the epidemic slowing down, we will be able to keep the incentive to interact with our customers during the pandemic, including making promotional messages on our communication software. From the store’s perspective, it can also be used for general greetings, etc., or even uploading served works, all of which can generate or promote consumer motivation and create their own business opportunities, a concept similar to a study done by Dwivedi, 2016 in.

This study is based on the content of the interview after the task operation. In the belief that the beauty industry is motivated by the demand, first of all, it is convenient and fast for most people, and the service design of the app is based on the concept of being people-oriented to facilitate access to services. Secondly, the establishment of trust that is the norm in the beauty industry services. Moreover, technology comes from the trend of demand, so the ability to handle online reservations or cancellations at any time is a very important feature of the booking platform. Finally, more service choices can trigger the motivation of consumers to use the app, including online reviews and price comparisons for all members of the platform as a reference. Therefore, the beauty industry service application app can strengthen the service orientation and deepen motive and intention.

#### Confirmation of Capacity, Opportunity, and Motivation to Influence Behavior

The 12 frameworks are integrated into the COM-B model theory of capabilities, opportunities, and motivations. The core purpose of conducting focus interviews is to identify the different dimensions of behavior change related to app platform users. From the analysis of the study, it was found that because of COVID-19 spread, people’s knowledgeability in crisis awareness knew that wearing masks, washing hands regularly and avoiding groups were self-protective behaviors. This also creates “no-touch” business opportunities. Interviewees can propose many creative service design methods because of the needs in the workplace. These self-management methods can allow platform users to easily and quickly find a match and even pay for the service to feel its worth. From the perspective of opportunity, operators can use the beauty industry’s three-party appointment service platform to book third-site services with consumer members. This can eliminate the concern of consumer member clustering, and make use of space and time. Those who have a physical storefront can also rent out the storefront space, creating a matchmaking income to create job opportunities. Therefore, the epidemic has brought about changes in the way of daily life, which can be accomplished through the app. This includes building or linking online shopping and online communication within the app platform to find more business opportunities in the system functions. Furthermore, the risk of infection and the sense of security is one of the psychological factors. Because people are generally aware of the channels of infection, more people will use tools to avoid contact with people. For example, the motivation of app users is safety and practicality. It also includes information about special offers, promotions, publications and service word-of-mouth, and celebrity recommendations. All of them will trigger the consumption motivation of consumers who cannot leave their homes during the epidemic. In turn, it will change the way more people consume and create more opportunities for other app platform users.

### Practical Suggestions

The COVID-19 outbreak has been spreading since the beginning of the outbreak. Various mutated variants of the virus are affecting human life around the world and the economy has been affected by the epidemic with unemployment getting worse. The topic of this study is the beauty industry app platform, which focuses on the beauty industry with different thinking to improve the service. After the operation of the app task, we can understand that the user habits completely overturn the working habits of consumers, designers, and stores, and are closer to real-life for the beauty industry. If we can make good use of consumers’ psychological needs to design service content and expand service fields, it will have a positive impact on this epidemic in society. According to the results of this study, the “Beauty Industry app Matching Mechanism Platform” was built after the confirmation of development requirements. This study proposes three practical recommendations for the future: 1. platform construction and development, 2. platform promotion methods, and 3. matching the government policy on the prevention of epidemics.

#### App Platform Marketing and Use

The overall economic benefits of using the platform are the result of various detailed plans during the app construction period. The benefits derived from the development of the platform system in this study will be closely related to the recruitment and promotion of user members. Therefore, in the promotion methods of creating brand topics, building awareness, and transmitting brand messages, we must make the expectations of platform users, such as suitable advertising spokespersons and high-intensity press conferences. Grasp the psychological factors of consumers in the wave of the spread of the epidemic, expand advertising app, and show that the beauty app platform is an anti-epidemic tool with safety.

#### Future App Platform Development and Integration Services

Due to the impact of the epidemic, all industries are in a recession. The government is trying to stabilize the economy and the epidemic by various means. Since beauty-related industries are close to people’s lives, and in order to avoid becoming a breach in the epidemic prevention, the function of OpenBeautiful in this study echoes the concept of the government and the epidemic fighting period. Therefore, we can strive to combine resources with the government for the public interest. We can also work with beauty industry groups to reach cooperation goals and create demand for market expansion.

#### Establishing Good Public Benefits With Government Departments Between Epidemic Prevention and Business

The app platform developed in this study can effectively integrate the basic shared needs of consumers, beauty industry technicians, and stores. However, in order to be accepted by consumers in the market, it is necessary to develop a WEB 2.0 design for the app platform, which should give more diverse choices to the consumer groups of app users. For example, we can link up with other social media for online beauty teaching. In addition to the general items in the existing platform, including postnatal care center and wedding services, we can also link up with medical beauty and obstetrics and gynecology centers to establish an app platform with a female consumer theme. In addition, the construction of the platform belongs to the peer-to-peer network mall, the realization of the app sharing economy, and the possibility of multi-functional business interests.

### Limitation and Future Direction

This study focuses on the OpenBeautiful app development requirements and invites four types of professionals, including engineers who develop app and users of OpenBeautiful app. There are three types of users, including general consumers, hairdressing technicians, and hairdressing stores. Through the COM-B model framework, this study developed appropriate interview questions based on the four dimensions of Capability, Opportunity, Motivation, and Behavior. After the focus group interviews, the interviews were transcribed verbatim, and the keywords were extracted from the conversations of the four interviewees to illustrate their feelings about the use of OpenBeautiful app development as a basis for future continuous development. This study is only for a specific app, OpenBeautiful app, to do the requirement confirmation, the practice and process of this study may not be generalized to other industry app development requirement confirmation. For future research development, other research methods, such as quantitative research methods, can be used to pinpoint the influencing factors of user behavior by targeting different attributes of users.”

## Data Availability Statement

The raw data supporting the conclusions of this article will be made available by the authors, without undue reservation.

## Author Contributions

Both authors listed have made a substantial, direct, and intellectual contribution to the work, and approved it for publication.

## Conflict of Interest

The authors declare that the research was conducted in the absence of any commercial or financial relationships that could be construed as a potential conflict of interest.

## Publisher’s Note

All claims expressed in this article are solely those of the authors and do not necessarily represent those of their affiliated organizations, or those of the publisher, the editors and the reviewers. Any product that may be evaluated in this article, or claim that may be made by its manufacturer, is not guaranteed or endorsed by the publisher.

## Appendix

**Appendix 1:** Comparison of single-factor and multi-factor models.

Project	Content description
General member management	User: FB login/download app login/line login Mobile phone number login/bind E-mail Forgot password Appointment record Order management
Store member management	Store member basic information/application contract Store classification management set/store technical staff management Store portfolio/product management Store member Store reservation/consultation management/order management Store venues open for appointment management Store-Technician-Financial Loss Return Form Complete the write-off code
Designer management	Technicians’ basic information/application contract documents Technical staff portfolio/commodity management set Technical staff appointment/consultation management/order management Reservation store venue/on-site service management Store-Technician-Financial Loss Return Form Complete the write-off code
Consumer management	Basic member information/application contract Search for stores/technicians/nearby locations Consulting system/message communication Add to Shopping Cart Make reservation system scheduling/checkout system Obtain consumer code Cancellation mechanism
Reservation system	Calendar appointment/time slot (consumer appointment system) (Store-technician venue reservation system)
Consulting system	Chatbox consulting system
Message system	Message reply system
Store (Designer) evaluation system	Star Rating/Number of Favorites/Number of Followers (Favorites)
Appointment management (Platform administrator)	Consumer member management/store management/technician management
Member Management (Platform administrator)	Consumer member management/store management/technical staff management/audit management system
Advertising management	Manufacturer/store/works display/marketing advertisement/public welfare message
SMS serial	Chunghwa Telecom SMS system serial connection
Electronic invoice (Platform Administrator)	MasterCard Electronic invoice concatenation
Online video teaching	Embed YouTube
Coupon management	Promotion code generation/net celebrity recommendation bonus (store/technician/consumer/platform administrator)
Write-off system	Code write-off (store/technician/consumer/platform administrator)
Order management system	Store (technician) order management system/administrator order management system
Connect payment flow system	MasterCard payment flow/credit card/super merchant code payment/ATM virtual account
Refund system	Order cancellation/refund mechanism
